# RNA Phage Biology in a Metagenomic Era

**DOI:** 10.3390/v10070386

**Published:** 2018-07-21

**Authors:** Julie Callanan, Stephen R. Stockdale, Andrey Shkoporov, Lorraine A. Draper, R. Paul Ross, Colin Hill

**Affiliations:** 1 APC Microbiome Ireland, University College Cork, Cork, T12 YT20, Ireland; julie.callanan@umail.ucc.ie (J.C.); s.stockdale@umail.ucc.ie (S.R.S.); andrey.shkoporov@ucc.ie (A.S.); l.draper@ucc.ie (L.A.D.); p.ross@ucc.ie (R.P.R.); 2 School of Microbiology, University College Cork, Cork, T12 YN60, Ireland; 3 Teagasc Food Research Centre, Moorepark, Fermoy, Cork, P61 C996, Ireland

**Keywords:** bacteriophage, RNA viruses, RNA, *Cystoviridae*, *Leviviridae*

## Abstract

The number of novel bacteriophage sequences has expanded significantly as a result of many metagenomic studies of phage populations in diverse environments. Most of these novel sequences bear little or no homology to existing databases (referred to as the “viral dark matter”). Also, these sequences are primarily derived from DNA-encoded bacteriophages (phages) with few RNA phages included. Despite the rapid advancements in high-throughput sequencing, few studies enrich for RNA viruses, i.e., target viral rather than cellular fraction and/or RNA rather than DNA via a reverse transcriptase step, in an attempt to capture the RNA viruses present in a microbial communities. It is timely to compile existing and relevant information about RNA phages to provide an insight into many of their important biological features, which should aid in sequence-based discovery and in their subsequent annotation. Without comprehensive studies, the biological significance of RNA phages has been largely ignored. Future bacteriophage studies should be adapted to ensure they are properly represented in phageomic studies.

## 1. Introduction

Bacteriophages, commonly known as “phages”, are the most abundant biological entities on the planet, with approximately 10^31^ in the biosphere [1]. Phages were independently identified in 1915 by Twort and in 1917 by d’Hérelle [2,3]. They are viruses which can alter microbial populations, with a major role in diversity patterns of microbial populations [4]. They were first recorded as antibacterial agents by d’Hérelle and quickly developed into clinical aids against bacterial infections, particularly across Eastern Europe [3,5,6]. The first known RNA phage, f2, which infects *Escherichia coli*, was described more than 40 years after the discovery of DNA phages [7]. Weissmann (1974), suggested that RNA phages offered a means to examine basic biological processes at an in-depth molecular level [8]. Since their identification, RNA phages have served as valuable models for understanding not just essential viral processes but also fundamental molecular mechanisms such as RNA genome replication, translational control, and gene regulation [9,10,11,12].

The RNA phage MS2, isolated by Alvin John Clark in 1961 and highly similar phage f2 [13], have become key models in molecular biology and genetics. The MS2 phage coat protein gene was the first gene to be completely sequenced in 1972 by Friers and his colleagues [14]. In addition, the genome of the MS2 phage was the first to be fully sequenced in 1976, also by Walter Friers and colleagues [15]. This preceded the sequencing of the first DNA based genome of phage phiX174 in 1977 [16]. RNA phages have also provided scientists with a model system for understanding the biology of many human pathogenic viruses such as Hepatitis, Influenza, and HIV [17,18,19]. For example, the stem loop structure of MS2 bacteriophage RNA has become a common tool for studying the key group antigen (Gag) polyprotein of HIV by replicating the protein–protein interactions [20].

While there is a substantial amount of literature and studies involving the bacterial component of the gut microbial system, there is still relatively little known about the human virome, and in particular the bacteriophage fraction of the microbiome. In addition, most newly identified phage sequences do not have known counterparts in viral databases, and these unknown sequences are sometimes referred to as the “viral dark matter” [1]. Phages influence microbial populations by infecting and destroying specific species of bacteria. Alternatively, temperate or lysogenic phage are capable of integrating their genomes into the host bacterium’s chromosome, often providing bacteria with a fitness advantage while the phage remains dormant and replicates in tandem with the host chromosome [21].

The genomic composition of phages are extremely diverse and are composed of either DNA or RNA, which can in turn be either single-stranded (ss) or double stranded (ds). Single-stranded RNA genomes can exist in two variants; negative sense (−ve) and positive sense (+ve). This depends on their orientation and whether there is a prerequisite for transcription prior to translation. Some eukaryotic RNA viruses use reverse transcriptase to replicate their genetic material through a DNA intermediate, while no DNA stage has been observed amongst bacterial RNA phages to date. In addition, the genomes of phages are described as being “mosaic”, composed of individual modules that may appear in other phages but in an alternative arrangement [22,23]. While phages can evolve through the accumulation of mutations, within environments they are responsible for vast amounts of genetic recombination and horizontal gene transfer events [24]. Altogether, these attributes make both DNA and RNA phage genomes very diverse and difficult to classify.

Typically, phage infection proceeds via adsorption, penetration, replication, assembly, and release. Briefly, phages use specialized surface receptor-binding proteins to interact with and adhere to their specific cognate host receptor. Phages then use various mechanisms to breach the cell wall of bacteria and inject their genomes into the cytoplasm of the host. Infection can then proceed via a lysogenic (temperate) or lytic (virulent) lifecycle. Virulent phages hijack the host’s cellular components to direct the replication of the phage’s genome and produce the necessary viral encoded proteins. Once the phage genome is replicated, it is packed into self-assembled viral particles. Phages induce host cell lysis and the assembled phage progeny are released into the surrounding environment for successive infections. Temperate phages that can replicate through lytic or lysogenic lifecycles are typically able to integrate into the bacterial chromosome and are subsequently replicated in tandem with the host genome. Temperate phages can also be maintained through formation of an episome within the host, which is disseminated through a population via cell division [25]. Temperate phages can respond to host cues from environmental stresses to initiate the lytic cycle and release phage progeny. They have been shown to dramatically affect susceptible bacterial populations through transfer of novel genes to their host, they can provide resistance to subsequent phage predation and can also alter host gene expression [26].

Although studies into the phage component of the microbiome have increased rapidly in recent years, databases are dominated by phages with DNA genomes. According to the latest (2017) report by the International Committee for the Taxonomy of Viruses (ICTV), viruses are separated into 134 families. The same report separated RNA phages into only two families; *Cystoviridae* (dsRNA phage) with 1 genus, *Cystovirus*, with 7 recognised species, and *Leviviridae* (ssRNA phage), with 2 genera, *Levivirus* and *Allolevivirus*, each of which contain two species [27,28].

A recent examination of RNA phage populations in 2016 by Krishnamurthy and Wang, through metagenomic dataset analysis, led to the identification of 122 partial genomes of novel RNA phages [29]. The host range of DNA phages typically varies greatly in contrast to that of RNA phages, which were all thought to target members of the Proteobacteria phylum. However in that study, an RNA phage was identified from a transcriptome of pure culture of *Streptomyces avermitilis*, a Gram-positive bacterium, known as *Streptomyces* phage phi0. This phage is thought to belong to the *Cystoviridae* family based on RNA-dependent RNA-polymerase (RdRp) analysis. This was the first report of an RNA phage with a natural affinity for a Gram-positive host. In addition, a recent pre-print has described an RNA virus, a planarian-infecting *Nidovirales*, with a genome of 41.1 kb in length, significantly longer than the previous largest RNA virus genome of 30 kb [30]. These findings highlight that there are certainly many RNA viruses yet to be discovered and described, including RNA phages.

This review focuses on examining known RNA phages, both dsRNA and ssRNA, which target bacterial cells. Outlined are their mechanisms of adsorption through to the release of progeny. Future endeavors may use conserved features of RNA phages as genetic signatures to aid in prospective metagenomic exploration of RNA phages in the “dark matter” via sequence-based targeting.

## 2. Cystoviridae

**Figure 1:**
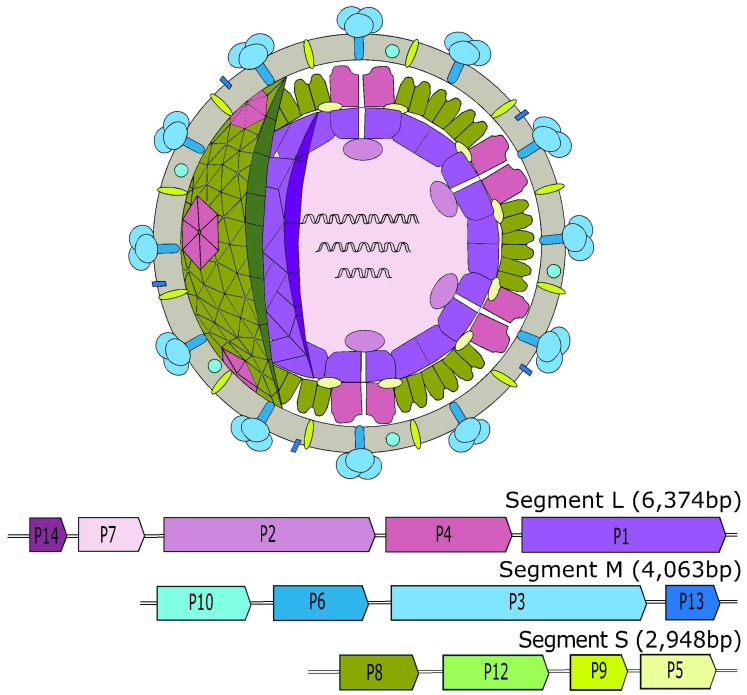
Virion of *Pseudomonas* phage phi6, the type virus of the *Cystoviridae* family. The virion and genes encoded by the tri-segmented genome of this phage are color co-ordinated. The grey circle represents the membrane encapsulating the virion. See text regarding gene information. (This figure was reproduced based on other images [28,32,33]).

Currently there are seven recognized species of *Cystoviridae* listed in the 2017 ICTV report. The type species of Cystovirus family is phi6, which for a long time was thought to be unique as a dsRNA phage. The *Cystoviridae* have a tri-segmented, linear dsRNA genome, with the concatenated genome varying size from 12.7 kb (phi2954) to 15.0 kb (phi8). Individual genome segments range in size from 2.9 kb to 6.4 kb (Figure 1). The three genome segments, large (L), medium (M) and small (S) are transcribed into separate polycistronic mRNAs that are predicted to be translated by the host machinery into 12 proteins. A lipid membrane envelops a double-layered proteinaceous nucleocapsid (NC) [31].

*Cystoviridae* genes are ordered into functional units within the segments: L-segment contains genes for the virion core (P1, P2, P4, and P7), the M-segment encodes the complex essential for host recognition (P3 and P6) and the S-segment is responsible for the shell protein of the nucleocapsid (P8 (except in phi8), P9, P12, and P5) [34,35,36,37,38,39,40,41,42]. P5 and P11 are transcript variants of the same gene [43]. The noncoding regions that flank the coding sequences within the segments are required for efficient genome replication and packaging. The 5′ untranslated region (UTR) of the plus strand region encodes a *cis*-acting RNA sequence known as the *pac* sequence [44]. The segment-specific *pac* sequence is composed of 200 nucleotides located within a number of stem-loop structures. The *pac* sequences act in unison with other fundamental structural elements to ensure the correct packaging of the genome when required.

The integral-membrane, fusogenic P6 protein is responsible for securing the receptor-binding protein of *Cystoviridae*, P3, to the viral envelope. It is this multimeric spike protein, P3, which enables the recognition of the host bacteria receptor pilin, the protein monomer making up bacterial pili, by the phi6 phage. The P3 protein of phages phi8, phi12, phi13, and phiYY have been suggested to be a single polypeptide or a multimer [45]. The P3 protein of phi6 adsorbs to host type IV pili of its target, *Pseudomonas syringae*, which then retracts to bring the phage into close proximity of the host membrane [46,47]. This form of attachment is also exploited by phiNN and phi2954 [37,40]. Other members of *Cystoviridae*, such as phi8, phi12, phi13, and phiYY, utilize their heteromeric P3 protein to attach to the lipopolysaccharide (LPS) on the cell surface [48]. The P3 protein of these species differs in its composition as it contains two or three different polypeptides (P3a, P3b and, in some cases, P3c). The P6 protein is activated following the removal of P3 and then mediates the fusion of the viral membrane with the host membrane to release the NC into the periplasmic space.

The loss of viral membrane around the NC enables the muralytic (peptidoglycan-degrading) enzyme P5, located on the NC surface, to degrade the peptidoglycan layer of the bacterial cell wall [49,50]. The permeabilization of the host plasma membrane facilitates the translocation of the NC across the cytoplasmic membrane of the host cell through an endocytosis-like process, driven by P8 [51,52]. Upon entry into the cytoplasm, the P8 shell of the NC dissociates to reveal the naked dodecahedral polymerase complex (PC). The release of P8 stimulates the PC, which is transcriptionally active. This is the characteristic mechanism dsRNA viruses exploit in order to replicate their genome—delivery of the nucleic acids in a specialized icosahedral capsule containing the necessary RNA metabolism enzymes such as mRNA synthesizing enzymes. This nano-compartment enables the dsRNA genome to remain “hidden” from any antiviral mechanisms of the host and avoids dsRNA induced host responses [53]. It also provides a safe environment for phage replication and translation. The dimeric P7 protein acts as an assembly and packaging cofactor by accelerating the rate of immature PC assembly through stabilization of the entire complex [54,55].

The core particle is composed of P1, P2, P4, and P7. These proteins are involved in the transcription of the phi6 genome. The monomeric RdRp of the P2 gene is activated by PC entry into the cytoplasm. This enzyme catalyzes the semi-conservative transcription of polycistronic mRNAs within the core particle [56]. Bacterial hosts lack the capability to synthesize complementary strands from the RNA template, so all characterized RNA viruses, including phages, encode their own enzymes. The RdRp attaches to the 3′ end of the single-stranded mRNA transcripts and through primer-independent de novo initiation it efficiently replicates and transcribes the phage genome [57,58]. The suggested transcription mechanism involves the dsRNA genome unwinding as it is pulled through one channel of P2 and nucleotide triphosphates (NTPs), oligonucleotides, manganese (Mn^2+^) and magnesium (Mg^2+^) ions entering through another [59]. Initially the template strand overextends and it locks into a “specificity pocket” [59]. The strand then reverses, in the presence of two cognate NTPs, to form the functional initiation complex. The reaction is primed through the activity of one of the NTPs as it serves as the carboxyl-terminal domain of the protein. It has been suggested that *Cystoviridae* control transcription through an interchange of two independent mechanisms (a) plus-sense initiation sites are preferred by the polymerase and (b) initiation competent ssRNA templates have more available transcription initiation sites [60]. Initiation is the rate-limiting step of transcription, located at the 3′-terminal cytidine nucleotide of the −ve ssRNA template.

By directly releasing the mRNA transcripts into the cytoplasm, the dsRNA genome is never exposed to the host cytoplasm which helps the phage to avoid host defense mechanism activation. The mRNA transcripts are used as templates for translation of the necessary proteins. The early stage of infection is characterized by equal amounts of mRNA from L, M, and S segments [61,62]. However, only the L-segment transcripts are efficiently produced in this early stage, to give rise to an increased level of PC proteins and the formation of empty PCs.

The large free-strand +ve ssRNA is then translated to form P1, P2, P4, and P7, which are subsequently assembled to form empty PCs [43]. The hexameric nucleoside triphosphatase (NTPase) motor of P4 directs the bundling of the three genome segments in the form of +ve ssRNA into the empty PCs by recognition of 5′ *pac* sequences [44,63]. This packaging is controlled through the expression of segment-specific binding sites on the PC. Binding sites specific for the S-segment are exposed initially to allow P4 to package the S-segment into the empty PC. A conformational change of the PC alters the binding site to become M-segment specific to package this segment of the genome into the viral progeny. Another change allows the packaging of the L-segment. Once the PC expands to a threshold size, these +ve ssRNA transcripts are then converted into dsRNA by a single round of negative strand synthesis of RdRp P2 [64]. Studies by Pirttimaa and colleagues (2002) found that of the 12 P4 hexamers, one is both functionally and structurally unique [65]. Although studies focused on the basic molecular mechanisms of phages have exploded in recent years, the exact transcriptional and translational processes of *Cystoviridae* are yet to be fully described in exact detail.

The size and organization of this PC is regulated through the activity of inner capsid protein P1 and P4 [55,66,67]. P1 is conserved throughout dsRNA viruses, although it appears to vary in multimeric status [55,68]. Transcription is initiated following effective replication of the dsRNA genome. As the infection progresses, the M and S segment mRNA predominate to produce the proteins essential to virion assembly. The naked PC is encapsulated in a newly synthesized NC shell. The membrane protein P9, along with morphogenic P12, have crucial roles in construction of a new phospholipid membrane around the NC particle from the host plasma membrane [69]. The spike protein complex of P3 and P6 is the last component attached to the surface, to ensure the progeny are capable of receptor recognition.

*Cystoviridae* are categorized as virulent phages as they induce lysis of their host bacterium at the end of the infection cycle in order to release viral progeny, through P5 and P10 activity [49,50]. However, recent findings have shown that phi6 is capable of forming a pseudolysogenic carrier state within its host [70]. *Cystoviridae* species phage phi6 targets the Gram-negative bacterium, *Pseudomonas syringae*, an important plant pathogen. This phage was first isolated in the 1970’s in the USA from *Pseudomonas-*infected bean straw [71].

There have recently been six additional *Cystoviridae* isolated and characterized in the 2017 ICTV report with another five requiring further analysis. Their genetic and structural similarities with phage phi6 suggest that there will be an expansion of this phage taxonomic family with further classification required. Sampling of various legumes in the USA have resulted in the isolation of additional dsRNA phages but these have not been characterized beyond their sequences [48,72,73]. Assorted environmental sources in Europe and Asia have yielded more novel dsRNA phages: *Pseudomonas* phage phiNN was isolated from a freshwater sample in Finland, while *Pseudomonas* phage phiYY came from hospital sewage waste in China [37,42]. Phage isolate phiYY has been found to target *P. aeruginosa* strains, an opportunistic pathogen of immuno-compromised individuals. This suggests there may be potential to develop a phage therapy to combat *Pseudomonas* infections in these individuals.

It is clear from the recent isolations of *Cystoviridae* from multiple environments, with only a single member infecting a Gram-positive host, that there are many more RNA phages yet to be discovered. Recently, Alphonse and Ghose (2017) examined known *Cystoviridae* using their encoded RdRp [32]. While ssRNA phage genomes have high mutation rates [74], RdRp appears to be conserved amongst RNA phage genomes and thus might be a good candidate as a genetic signature to identify further RNA phage sequences. However, identification of *Cystoviridae* in metagenomic datasets using a marker such as the RdRp is complicated by the tri-segmented nature of the *Cystoviridae* genomes. Therefore, sequence based detection of all three genomic segments of *Cystoviridae*, particularly if they are divergent from sequences present in public repositories, will be challenging. Incorporation of genetic tags from each of the three segments will greatly enhance de novo efforts of finding *Cystoviridae* members.

## 3. Leviviridae

**Figure 2:**
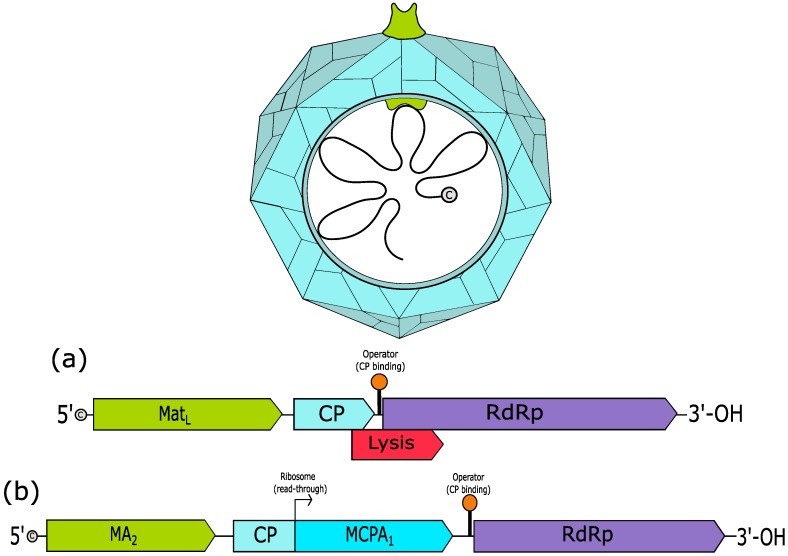
Virion of typical *Leviviridae* family. (**a**) Genome of Enterobacteria phage MS2, an example of a *Levivirus* (3569 bp)*.* (**b**) Genome of Enterobacteria phage Qß, an example of an *Allolevivirus* (4215 bp)*.* The genomes and the virion structures are color-coded. (Mat_L_: maturation protein of *Levivirus*, MA_2_: maturation protein A_2_ of *Allolevivirus*, CP: coat protein, MCPA_1_: minor-CP A_1_ of *Allolevivirus*, RdRp: RNA-dependent RNA polymerase) (These figures were created based on a previous depiction [27,75]).

The *Leviviridae* family encompass phages with a positive-sense single stranded, monopartite RNA genome of 3.3–4.3 kb in length. The nonenveloped, somewhat spherical virion capsid is composed of 178 copies of the dimeric coat protein (CP) and a single copy of the maturation protein (Figure 2). The 5′ end of the genome carries a triphosphate cap.

There are two genera of *Leviviridae*; the *Levivirus* and *Allolevivirus*. These genera were historically differentiated through serological cross-reactivity, sedimentation, molecular weight and density [76]. More recently, the number of known genes in their genomes have been used to distinguish between *Levivirus* and *Allolevivirus* members, with three and four, respectively (Figure 2). These genera are subdivided into genogroups; *Levivirus* has MS2-like (genogroup I) and BZ13-like (genogroup II) and *Allolevivirus* has Qß-like (genogroup III) and F1-like (genogroup IV) [27].

*Leviviridae* phages that target *E. coli*, “coliphage”, which are male-specific, adsorb along the fertility (F) pilus, coded by the F-plasmid of *Escherichia coli*, or the chromosomal marker Hfr, whereas in non-coliphage species alternative pili are exploited [77]. Alternatively, coliphages that can infect cells via the cell wall are classified as somatic [78]. The presence of enteroviruses in water from pollution is often detected through the identification of RNA coliphages as biomarkers [79]. Phages that utilize F-pili are classified as male-specific phages. The way in which the *Leviviridae* phages induce lysis of their host is a notable difference between the genera; *Levivirus* phages encode a separate lysis polypeptide, whereas *Allolevivirus* phages utilise their maturation protein in lysis mediation [80,81]. These proteins are two canonical “single gene lysis” (SGL) systems that are utilised by small phages, the third is the *E* lysin from phage φX174, a ssDNA *Microviridae* representative [82]. The lysis mechanism, and specific protein where applicable, is fundamental to the lifecycle of the phage.

### 3.1. Levivirus

The type species of *Levivirus* is the *Enterobacteria* phage MS2, a member of the MS2-like phages (genogroup I). Phages of the *Levivirus* genus infect their host targets through the initial adsorption of the virion along the sides of pili using the “maturation A-protein” (Mat_L_) as the receptor binding protein [83]. This results in the self-proteolytic cleavage of the A-protein into at least two fragments and a structural change of the F-pilus [84]. This induces the release of the phage RNA into the host bacterium. Studies have reported that the two largest polypeptide components are transferred into the host along with the genomic RNA [84]. The fragmented Mat_L_ binds the RNA at two distinct regions: the Mat_L_ coding region and the 3′-UTR [85]. It appears that Mat_L_-RNA complex may be injected into the cell as opposed to free RNA, suggesting that the Mat_L_ protein may have a greater biological role than originally envisaged [84]. The exact mechanism of how the Mat_L_-RNA complex gains entry to the host remains undescribed, but could involve a type IV secretion system (T4SS) homolog [86]. It has been postulated that Mat_L_ may also contribute to the replication process of the RNA genome.

As the nucleic acid is a single copy of +ve ssRNA, it functions both as the genome template and mRNA upon infection. Thus, there is constant competition between replication and translation processes as the ribosome and replicase run in opposite directions along the template strand [87]. The two events are independent of each other with the secondary structures of the +ve ssRNA strand and formation of a complementary negative strand of RNA maintaining this equilibrium. It has been noted that in the 3′-terminal sequence of the *Levivirus* genomes, there is a signature sequence of 5′-ACCACCCA-3′ [88].

For effective genome replication, *Leviviruses* encode a copy of RdRp that codes for the catalytic ß-subunit of the replicase. This protein associates with three host proteins: ribosomal protein S1 [89] and the translational elongation factors EF-Tu and EF-Ts [90], to form a functional polymerase unit, the holoenzyme. The role of EF-Tu has been established as delivering an aminoacyl-tRNA to the ribosome when in its GTP-bound form [91]. This GTP is hydrolyzed to form GDP-bound EF-Tu following a codon anti-codon match within the ribosomal complex. This displaces the EF-Tu and EF-Ts binds to the GDP-bound EF-Tu and removes the GDP molecule. This allows the EF-Tu to be recycled for further elongation rounds [92,93]. Sequestration of these elongation factors inhibits initiation of translation. The S1 protein functions as a translational initiation factor. The sole purpose of this protein is to recognize the template plus strand, the core-complex of the three remaining proteins is sufficient to synthesize new +ve ssRNA strands [94].

Studies have shown that there are two internal sequences which are key to the recognition of the plus strand by the replicase, the S site and M site. [95]. The S site is described as being a uracil rich sequence of approximately 100 nucleotides, located just before the initiation codon of the coat protein. The secondary structure of the S site is poorly defined. The M site is of similar length, forms a branched stem-loop structure and resides within the replicase coding region [96]. These two sites are simultaneously bound by the S1 protein to allow for effective replication by the replicase through enhanced recognition of the template to the active site [97].

The RNA template is protected from cellular nuclease degradation through an unknown mechanism. There is an additional host factor required for successful translation that has been isolated but not genetically identified in the case of *Levivirus* species. This protein does not interact with the polymerase machinery but instead binds directly to the 3′ terminal of the mRNA template [98]. The replicase will associate to the start site and initiate negative-strand synthesis by replicating through the genome. This strand is used to synthesize new +ve ssRNA genomes for the viral progeny.

As the infection cycle reaches the end-stage, the CP-dimers bind the replicase gene start site, located within a hairpin-structured operator, and act as translational repressors [99,100]. This results in a packaging signal that stimulates the assembly of functional viral progeny. At the same time, there is an increase in quantities of the lysis protein, with a single lysis protein required for each phage progeny. Since the lysis protein lyses the cell without affecting the integrity of the peptidoglycan network, and in the absence of muralytic enzyme activity, it is referred to as an “amurin” [101]. Research focused on this protein has revealed that it is primarily localized in Bayer’s patches, the periplasmic zones of adhesion between the inner and outer membrane [102]. The exact mechanism by which this 75-amino acid lysin induces host lysis is not exactly known [103]. However, the current proposal is that the lysis protein forms lesions and hydrophobic pores in the inner membrane that dissipates the proton motive force (PMF) [104]. This alteration in PMF activates autolysis of the bacterial host through certain enzymes such as DD-endopeptidases and lytic transglycosylases. Supporting research has shown alteration in the average length in glycan strands and degree of cross-linkage, suggesting the activation of the aforementioned enzymes [105].

Nonetheless, the molecular information and functioning schema of such an autolytic pathway have yet to be identified [82]. Recent findings have indicated that the lysis of host cells by MS2 lysin is dependent on a range of host factors, including host chaperone DnaJ [106]. This post-translational regulator allows for another level of control of both quantity and activity of the lysis protein of MS2.

Translation of the phage genes requires the ribosome to associate with the RNA through a Shine–Dalgarno sequence, the start codon and the host ribosomal S1 protein. The S1 protein can bind the S site, as mentioned above. This creates a situation whereby the S1 protein of the ribosome and replicase are competing for the same RNA binding site.

There are a variety of systems that regulate protein synthesis, including: RNA secondary structure, ribosome access to the initiation codon, and folding kinetics [107,108,109]. The secondary structures of the +ve ssRNA are the predominant factors in determining different protein yields; e.g., the CP gene is free from any secondary structures as it is required in high copy numbers (178 per virion), whereas the replicase gene is trapped in tight secondary structures as only one copy per progeny is required [110]. The open reading frame (ORF) of the coat protein is readily available for the ribosomal translation. As the ribosome moves along the RNA transcript, it disrupts the secondary structure to allow hidden genes to be translated. Following CP gene translation, the initiation codon of the replicase gene becomes available, resulting in the synthesis of the replicase ß-catalytic subunit. The translation of the lysis and replicase gene is dependent on successful translation of the CP gene.

Newly synthesized viral particles require only one copy of the lysis protein and the Mat_L_ protein [111,112,113]. The ORF of the lysis gene overlaps the replicase gene in a +1 frameshift, with the termination sequence of the lysis protein located in the coding region of the replicase gene [103,114]. Studies by van Duin and his colleagues (1990) on the translational control of the lysis protein provided key information as to the role of secondary structures in transcriptional regulation. Their work demonstrated that the formation of a stable hairpin in the RNA between the Shine–Dalgarno sequence and the start codon of the lysis gene, represses the expression of the lysis gene. Following successful transcription, during translation there is incomplete dissociation of the ribosome from the mRNA as it creates the CP protein [115,116]. The ribosome backtracks to reinitiate at the start codon for the lysis protein in approximately 5% of translational cycles [117,118]. The lysis protein is produced at low levels towards the ends of the infection cycle. This allows for gradual accumulation of the lysis protein to ensure that the viral progeny have sufficient time to mature.

The Mat_L_ protein is only transcribed from newly synthesized genome templates [119]. The strong secondary structure formed by the Shine–Dalgarno sequence and the S1-binding sequence prevent translation of the 5′-end in normal mRNA structure, where the Mat_L_ gene is positioned. In nascent RNA strands, there is an alternate, shorter hairpin structure created that enables translation of the maturation gene in the 5′ terminal by allowing access and binding of the ribosome. This RNA-folding intermediate of newly-synthesized strands enable the ribosome access to the start codon of the A-protein.

A recently isolated RNA phage for *Acinetobacter* species, AP205, was found to have an unusual genome structure with the lysis gene located in the 5′ terminal [120]. Although the genome of AP205 mirrors the typical *Levivirus* genome map, the secondary structure and 3′-UTR follows that of *Allolevivirus.* This phage has yet to be approved as a *Levivirus.* Potential *Leviviruses* of *Pseudomonas*, phages PPR7 and PRR1, have also been isolated and characterized, both exhibit particular hallmarks of *Levivirus* phages [121,122,123,124].

### 3.2. Allolevirus

The type species of *Allolevivirus* is Qß, the representative of the Qß-like phages (genogroup III). Species of *Allolevivirus* contain a longer version of the genome with an extension of the C-terminal of the CP gene [76]. The presence of this minor-CP A_1_ (MCPA_1_) protein, also known as the ‘read-through protein’, is a feature unique to *Allolevivirus* phages [125]. Both the MCPA_1_ and the maturation A_2_ (MA_2_) proteins are essential for host attachment [76]. The majority of *Allolevivirus* members were found to encode an Arg-Gly-Asp (RGD) motif, essential for host cell recognition and attachment, within their MCPA_1_ and/or MA_2_ [88]. This motif is absent in the *Levivirus* phages. Similar to the signature 3′-terminal sequence of *Levivirus*, *Allolevivirus* species contain a 5′-TCCTCCCA-3′ within the 3′-terminal of their genome [88].

The underlying translation and replication mechanisms are similar to *Levivirus* with minor variations. The host factor that associates with the functional replicase has been isolated, purified and genetically characterized for Qß as the protein encoded by the host factor of Qß (*hfq*) gene of *E. coli* [126,127]. This nonspecific ssRNA binding protein, Hfq, aids polymerase association to the 3′ end of the +ve ssRNA template. The start of the 5′-terminal begins with a GG sequence. There is a nontranslated A residue attached to the extreme 3′ terminus in a CCA sequence, following activity of the terminal nucleotidyl transferase (TNTase) domain of RdRp [128,129,130]. This does not serve as a template nucleotide, instead RNA synthesis begins at the penultimate C residue.

The transition between replication and translation is similar to the above mentioned *Levivirus*, with a slight change; the translation of the (MA_2_) is controlled in a temporal manner as opposed to a structural intermediate. This is dictated by the length of time it takes for the polymerase to move from the start site of the maturation gene to the complement of the Shine–Dalgarno sequence [131,132]. Once it has been translated, these two sequences bind to form a strong secondary structure to prevent continuous translation of the same gene. The additional MCPA_1_ protein is formed following ribosomal read-through of the leaky-stop codon (UGA) of the CP gene [118]. It is read as a tryptophan codon (UGG), which promotes gene expression of the MCPA_1_ protein. The ribosome occasionally, in approximately 5% of cases, translates past this leaky termination sequence for an additional 600 nucleotides to form a C-terminal extension of the CP [133]. This protein is incorporated in low quantities into viral progeny and is essential for successful infection. Studies of the amino acid sequence and the three-dimensional structure of the MCPA_1_ protein, have shown it to be unique to the small group of *Allolevivirus* phages [133]. The MA_2_ and the MCPA_1_ protein, whose exact role is unknown as of yet, are essential for successful infection of pili-positive hosts

Another notable difference in the infection pattern of *Allolevivirus* is the absence of a lysis gene in the genome. Instead, the MA_2_ protein has a secondary function to induce the lysis of the host cell for release of viral progeny [134]. The MA_2_ protein is referred to as an amurin as it does not destroy the peptidoglycan layer directly through muralytic activity. It is also known as an “antibiotic protein” due to the similarity in function to antibacterial agents which target cell walls [135]. It has been reported that MA_2_ induces host cell lysis by inhibiting the enzymatic activity of MurA, a UDP-*N*-acetylglucosamine-enolpyruvyl transferase. This is an essential enzyme in the production of peptidoglycan as it catalyses the first committed step, the biosynthesis of murein precursor [136]. At the next stage of cell division, the inhibition of cell wall biosynthesis leads to host lysis and release of the phage progeny.

A study by Friedman et al. (2009), noted that the sequences of both *Levivirus* and *Allolevivirus* genera had strong homogeny across position of ORF, length of proteins and the catalytic ß-domains of the RdRp [88]. The conservation of the YGDD motif of the replicase protein across all positive-sense ssRNA viruses was recorded throughout the *Leviviridae.*

Although both *Levivirus* and *Allolevivirus* phages target the pilus of their hosts as receptors to initiate, the fact there is no conserved infection mechanism suggests that there may be varying mechanisms for the RNA to enter the cell. Originally thought to only affect plasmid-encoded appendages, there have been *Leviviridae* specific for genome-encoded pili of Gram-negative bacteria, such as *Pseudomonas* phage PP7 and *Acinetobacter* phage AP205.

## 4. Discussion

Although there have only been a limited number of RNA phages identified to date, their “true” diversity and abundance in nature remains unknown. Current approaches used for the isolation, selection, and purification of viral particles, including precipitation by polyethylene glycol (PEG) and caesium-chloride (CsCl) gradient purification, are almost certainly biased against RNA phages [137]. The selection of DNA phages in these methods goes a long way to explaining why RNA phages are under-represented in genome databases.

The fragile nature of RNA and the widespread presence of RNases in human and animal derived samples also hinders studies involving RNA phages. The development of RNA phage-selective isolation protocols will also greatly enhance our endeavors. For example, separation of DNA and RNA fractions of samples and complete eradication of unwanted RNase is recommended. It should also be noted that the low abundance of RNA phages in databases will result in reduced “hits” for novel sequences. As research into the RNA section of the phage community is expanded, we can expect the databases to become more representative of the wider RNA phage community. 

An interesting paper recently proposed that members of the *Picobirnaviridae* family may not be eukaryotic viruses as originally thought, but may in fact represent a novel family of RNA phages [138]. This research involved analysis of bacterial ribosome binding sites (RBS) upstream of the coding sequences in their bi-segmented, dsRNA genomes. It was noted that an RBS motif, thought to be unique to prokaryotic-infecting viruses, was enriched in the picobirnaviruses. This finding suggests that these dsRNA viruses could be classified as putative bacteriophages. Furthermore, an additional study has supported this hypothesis by proposing that picobirnaviruses are in fact a novel RNA phage family of high genomic diversity [139]. This type of analysis demonstrates the possibility that more members of RNA virus populations may in fact be mischaracterized. A more robust method for classification of RNA phages would help to resolve this issue.

Identifiable RNA phage-specific domains, such as the RdRp gene, capsid gene, maturation protein gene, or the NTPase gene, can serve as features which one could use to mine metagenomic databases for RNA phages. However, since the RdRp gene is conserved amongst RNA viruses, unique genetic elements of *Leviviridae* and *Cystoviridae* families should also be used in specific studies. Contigs with homologs to both the leviviral and cystoviral RdRp gene are potential RNA phages and should be subjected to further analysis. Based on the recent studies mentioned above, homologs to the RdRp gene of picobirnaviruses should also be included [138]. The study by Krishnamurthy and colleagues which identified 20 unique RNA phage phylotypes utilized nucleotide identity to the RdRp and the maturation gene to categorize these phages [29]. The specific 3′-terminal sequences of *Levivirus* and *Allolevivirus* members could be used to further classify these phages. Signature features of *Cystoviridae* members, such as the muralytic enzyme gene or the nucleocapsid shell protein gene, could also serve as genetic signatures when screening the databases for RNA phages [42].

A common theme of this review is the need for greater efforts to be directed towards the discovery of more RNA phages for all potential applications, such as tools for advancing molecular biology and as potential phage therapeutics. The rise in antimicrobial resistance across bacteria is not a novel problem but it is alarming. The host range of RNA phages could offer therapeutic potential against some of the World Health Organizations’ (WHO) list of deadly pathogens, including some of the Gram-negative members of the ESKAPE pathogens, such as *Klebsiella pneumoniae*, *Acinetobacter baumannii*, and *Pseudomonas aeruginosa*. Clinical isolates of *P. aeruginosa* have been found to be resistant to most of the antibiotics normally used to treat this infection [140]. An unclassified *Levivirus P. aeruginosa* phage PP7 has been identified which targets this bacterium via a pilin-specific mechanism [141]. Further studies regarding the therapeutic parameters of RNA phages, such as PP7, should be done to examine their efficiency to control these pathogens and to explore their potential use as components of cocktails used in phage therapy.

## References

[1] Hatfull G.F. (2015). Dark Matter of the Biosphere: The Amazing World of Bacteriophage Diversity. J. Virol..

[2] Twort F.W. (1915). An Investigation on the nature of ultra-microscopic viruses. Lancet.

[3] d’Herelle F. (1917). Sur un microbe invisible antagoniste des bacilles dysentériques. CR Acad. Sci. Paris.

[4] Rodriguez-Valera F., Martin-Cuadrado A.-B., Rodriguez-Brito B., Pašić L., Thingstad T.F., Rohwer F., Mira A. (2009). Explaining microbial population genomics through phage predation. Nat. Rev. Microbiol..

[5] Carlton R. (1999). Phage Therapy: Past History and Future Prospects. Arch. Immunol. Ther. Exp.-Engl. Ed..

[6] Summers W.C. (2012). The strange history of phage therapy. Bacteriophage.

[7] Loeb T., Zinder N.D. (1961). A bacteriophage containing RNA. Proc. Natl. Acad. Sci. USA.

[8] Weissmann C. (1974). The making of a phage. FEBS Lett..

[9] Brown D., Gold L. (1996). RNA replication by Q beta replicase: A working model. Proc. Natl. Acad. Sci. USA.

[10] Gytz H., Mohr D., Seweryn P., Yoshimura Y., Kutlubaeva Z., Dolman F., Chelchessa B., Chetverin A.B., Mulder F.A.A., Brodersen D.E. (2015). Structural basis for RNA-genome recognition during bacteriophage Qβ replication. Nucleic Acids Res..

[11] Lodish H.F. (1968). Bacteriophage f2 RNA: Control of Translation and Gene Order. Nature.

[12] Stock-Ley P.G., Stonehouse N.J., Valegård K. (1994). Molecular mechanism of RNA phage morphogenesis. Int. J. Biochem..

[13] Davis J.E., Strauss J.H., Sinsheimer R.L. (1961). Bacteriophage MS2: Another RNA Phage. Science.

[14] Jou W.M., Haegeman G., Ysebaert M., Fiers W. (1972). Nucleotide Sequence of the Gene Coding for the Bacteriophage MS2 Coat Protein. Nature.

[15] Fiers W., Contreras R., Duerinck F., Haegeman G., Iserentant D., Merregaert J., Min Jou W., Molemans F., Raeymaekers A., Van den Berghe A. (1976). Complete nucleotide sequence of bacteriophage MS2 RNA: Primary and secondary structure of the replicase gene. Nature.

[16] Sanger F., Air G.M., Barrell B.G., Brown N.L., Coulson A.R., Fiddes J.C., Iii C.A.H., Slocombe P.M., Smith M. (1977). Nucleotide sequence of bacteriophage φX174 DNA. Nature.

[17] Adcock N.J., Rice E.W., Sivaganesan M., Brown J.D., Stallknecht D.E., Swayne D.E. (2009). The use of bacteriophages of the family Cystoviridae as surrogates for H5N1 highly pathogenic avian influenza viruses in persistence and inactivation studies. J. Environ. Sci. Health Part A.

[18] Kenyon J.C., Prestwood L.J., Lever A.M.L. (2015). A novel combined RNA-protein interaction analysis distinguishes HIV-1 Gag protein binding sites from structural change in the viral RNA leader. Sci. Rep..

[19] Wang S., Liu Y., Li D., Zhou T., Gao S., Zha E., Yue X. (2016). Preparation and evaluation of MS2 bacteriophage-like particles packaging hepatitis E virus RNA. FEMS Microbiol. Lett..

[20] Becker J.T., Sherer N.M. (2017). Subcellular Localization of HIV-1 gag-pol mRNAs Regulates Sites of Virion Assembly. J. Virol..

[21] Harrison E., Brockhurst M.A. (2017). Ecological and Evolutionary Benefits of Temperate Phage: What Does or Doesn’t Kill You Makes You Stronger. BioEssays.

[22] Vasiljeva I., Kozlovska T., Cielens I., Strelnikova A., Kazaks A., Ose V., Pumpens P. (1998). Mosaic Qβ coats as a new presentation model. FEBS Lett..

[23] Hatfull G.F. (2008). Bacteriophage Genomics. Curr. Opin. Microbiol..

[24] De la Cruz F., Davies J. (2000). Horizontal gene transfer and the origin of species: Lessons from bacteria. Trends Microbiol..

[25] Cenens W., Makumi A., Govers S.K., Lavigne R., Aertsen A. (2015). Viral Transmission Dynamics at Single-Cell Resolution Reveal Transiently Immune Subpopulations Caused by a Carrier State Association. PLoS Genet..

[26] Howard-Varona C., Hargreaves K.R., Abedon S.T., Sullivan M.B. (2017). Lysogeny in nature: Mechanisms, impact and ecology of temperate phages. ISME J..

[27] Olsthoorn R.C.L., Van Duin J. Leviviridae—Positive Sense RNA Viruses—Positive Sense RNA Viruses (2011)—International Committee on Taxonomy of Viruses (ICTV). https://talk.ictvonline.org/ictv-reports/ictv_9th_report/positive-sense-rna-viruses-2011/w/posrna_viruses/263/leviviridae.

[28] Poranen M.M., Mäntynen S. (2017). ICTV Virus Taxonomy Profile: Cystoviridae. J. Gen. Virol..

[29] Krishnamurthy S.R., Janowski A.B., Zhao G., Barouch D., Wang D. (2016). Hyperexpansion of RNA Bacteriophage Diversity. PLoS Biol..

[30] Saberi A., Gulyaeva A.A., Brubacher J., Newmark P.A., Gorbalenya A. (2018). A planarian nidovirus expands the limits of RNA genome size. bioRxiv.

[31] Van Etten J., Lane L., Gonzalez C., Partridge J., Vidaver A. (1976). Comparative Properties of Bacteriophage φ6 and φ6 Nucleocapsid. J. Virol..

[32] Alphonse S., Ghose R. (2017). Cystoviral RNA-directed RNA polymerases: Regulation of RNA synthesis on multiple time and length scales. Virus Res..

[33] Viral Zone: Cystoviridae. https://viralzone.expasy.org/165?outline=all_by_species.

[34] Gottlieb P., Wei H., Potgieter C., Toporovsky I. (2002). Characterization of φ12, a Bacteriophage Related to φ6: Nucleotide Sequence of the Small and Middle Double-Stranded RNA. Virology.

[35] Gottlieb P., Metzger S., Romantschuk M., Carton J., Strassman J., Bamford D.H., Kalkkinen N., Mindich L. (1988). Nucleotide sequence of the middle dsRNA segment of bacteriophage φ6: Placement of the genes of membrane-associated proteins. Virology.

[36] Hoogstraten D., Qiao X., Sun Y., Hu A., Onodera S., Mindich L. (2000). Characterization of φ8, a Bacteriophage Containing Three Double-Stranded RNA Genomic Segments and Distantly Related to φ6. Virology.

[37] Mäntynen S., Laanto E., Kohvakka A., Poranen M.M., Bamford J.K.H., Ravantti J.J. (2015). New enveloped dsRNA phage from freshwater habitat. J. Gen. Virol..

[38] McGraw T., Mindich L., Frangione B. (1986). Nucleotide sequence of the small double-stranded RNA segment of bacteriophage phi 6: Novel mechanism of natural translational control. J. Virol..

[39] Mindich L., Nemhauser I., Gottlieb P., Romantschuk M., Carton J., Frucht S., Strassman J., Bamford D.H., Kalkkinen N. (1988). Nucleotide sequence of the large double-stranded RNA segment of bacteriophage phi 6: Genes specifying the viral replicase and transcriptase. J. Virol..

[40] Qiao X., Sun Y., Qiao J., Sanzo F.D., Mindich L. (2010). Characterization of F2954, a newly isolated bacteriophage containing three dsRNA genomic segments. BMC Microbiol..

[41] Qiao X., Qiao J., Onodera S., Mindich L. (2000). Characterization of φ13, a Bacteriophage Related to φ6 and Containing Three dsRNA Genomic Segments. Virology.

[42] Yang Y., Lu S., Shen W., Zhao X., Shen M., Tan Y., Li G., Li M., Wang J., Hu F. (2016). Characterization of the first double-stranded RNA bacteriophage infecting *Pseudomonas aeruginosa*. Sci. Rep..

[43] Carpino J. (2014). Structure and Function in Bacteriophage Phi6. Ph.D. Thesis.

[44] Mindich L. (1999). Precise Packaging of the Three Genomic Segments of the Double-Stranded-RNA Bacteriophage φ6. Microbiol. Mol. Biol. Rev..

[45] Mäntynen S., Sundberg L.-R., Poranen M.M. (2018). Recognition of six additional cystoviruses: Pseudomonas virus phi6 is no longer the sole species of the family *Cystoviridae*. Arch. Virol..

[46] Bamford D.H., Palva E.T., Lounatmaa K. (1976). Ultrastructure and Life Cycle of the Lipid-containing Bacteriophage φ6. J. Gen. Virol..

[47] Roine E., Raineri D.M., Romantschuk M., Wilson M., Nunn D.N. (1998). Characterization of Type IV Pilus Genes in Pseudomonas syringae pv. tomato DC3000. Mol. Plant-Microbe Interact..

[48] Mindich L., Qiao X., Qiao J., Onodera S., Romantschuk M., Hoogstraten D. (1999). Isolation of Additional Bacteriophages with Genomes of Segmented Double-Stranded RNA. J. Bacteriol..

[49] Mindich L., Lehman J. (1979). Cell Wall Lysin as a Component of the Bacteriophage ø6 Virion. J. Virol..

[50] Caldentey J., Bamford D.H. (1992). The lytic enzyme of the Pseudomonas phage φ6. Purification and biochemical characterization. Biochim. Biophys. Acta BBA—Protein Struct. Mol. Enzymol..

[51] Poranen M.M., Daugelavičius R., Ojala P.M., Hess M.W., Bamford D.H. (1999). A Novel Virus–Host Cell Membrane Interaction: Membrane Voltage–Dependent Endocytic-like Entry of Bacteriophage φ6 Nucleocapsid. J. Cell Biol..

[52] Romantschuk M., Olkkonen V.M., Bamford D.H. (1988). The nucleocapsid of bacteriophage phi 6 penetrates the host cytoplasmic membrane. EMBO J..

[53] Poranen M.M., Bamford D.H. (2012). Assembly of Large Icosahedral Double-Stranded RNA Viruses. Viral Molecular Machines.

[54] Juuti J.T., Bamford D.H. (1997). Protein P7 of phage phi6 RNA polymerase complex, acquiring of RNA packaging activity by in vitro assembly of the purified protein onto deficient particles. J. Mol. Biol..

[55] Poranen M.M., Paatero A.O., Tuma R., Bamford D.H. (2001). Self-Assembly of a Viral Molecular Machine from Purified Protein and RNA Constituents. Mol. Cell.

[56] Usala S.J., Brownstein B.H., Haselkorn R. (1980). Displacement of parental RNA strands during in vitro transcription by bacteriophage φ6 nucleocapsids. Cell.

[57] Blumenthal T. (1980). Qbeta replicase template specificity: Different templates require different GTP concentrations for initiation. Proc. Natl. Acad. Sci. USA.

[58] Silverman P.M. (1973). Replication of RNA viruses: Specific binding of the Qβ RNA polymerase to Qβ RNA. Arch. Biochem. Biophys..

[59] Butcher S.J., Grimes J.M., Makeyev E.V., Bamford D.H., Stuart D.I. (2001). A mechanism for initiating RNA-dependent RNA polymerization. Nature.

[60] Yang H., Makeyev E.V., Butcher S.J., Gaidelyte A., Bamford D.H. (2003). Two Distinct Mechanisms Ensure Transcriptional Polarity in Double-Stranded RNA Bacteriophages. J. Virol..

[61] Coplin D.L., Etten J.L.V., Koski R.K., Vidaver A.K. (1975). Intermediates in the biosynthesis of double-stranded ribonucleic acids of bacteriophage phi 6. Proc. Natl. Acad. Sci. USA.

[62] Emori Y., Iba H., Okada Y. (1983). Transcriptional regulation of three double-stranded RNA segments of bacteriophage phi 6 in vitro. J. Virol..

[63] Frilander M., Bamford D.H. (1995). In VitroPackaging of the Single-stranded RNA Genomic Precursors of the Segmented Double-stranded RNA Bacteriophage ψ: The Three Segments Modulate Each Other’s Packaging Efficiency. J. Mol. Biol..

[64] Poranen M.M., Tuma R., Bamford D.H. (2005). Assembly of Double-Stranded RNA Bacteriophages. Advances in Virus Research.

[65] Pirttimaa M.J., Paatero A.O., Frilander M.J., Bamford D.H. (2002). Nonspecific Nucleoside Triphosphatase P4 of Double-Stranded RNA Bacteriophage 6 Is Required for Single-Stranded RNA Packaging and Transcription. J. Virol..

[66] Qiao J., Qiao X., Sun Y., Mindich L. (2003). Isolation and Analysis of Mutants of Double-Stranded-RNA Bacteriophage φ6 with Altered Packaging Specificity. J. Bacteriol..

[67] Qiao X., Qiao J., Mindich L. (2003). Analysis of Specific Binding Involved in Genomic Packaging of the Double-Stranded-RNA Bacteriophage φ6. J. Bacteriol..

[68] Kainov D.E., Butcher S.J., Bamford D.H., Tuma R. (2003). Conserved Intermediates on the Assembly Pathway of Double-stranded RNA Bacteriophages. J. Mol. Biol..

[69] Stitt B.L., Mindich L. (1983). Morphogenesis of bacteriophage phi 6: A presumptive viral membrane precursor. Virology.

[70] Onodera S., Olkkonen V.M., Gottlieb P., Strassman J., Qiao X.Y., Bamford D.H., Mindich L. (1992). Construction of a transducing virus from double-stranded RNA bacteriophage phi6: Establishment of carrier states in host cells. J. Virol..

[71] Vidaver A.K., Koski R.K., Etten J.L.V. (1973). Bacteriophage φ6: A Lipid-Containing Virus of Pseudomonas phaseolicola. J. Virol..

[72] O’Keefe K.J., Silander O.K., McCreery H., Weinreich D.M., Wright K.M., Chao L., Edwards S.V., Remold S.K., Turner P.E. (2010). Geographic Differences in Sexual Reassortment in Rna Phage. Evolution.

[73] Silander O.K., Weinreich D.M., Wright K.M., O’Keefe K.J., Rang C.U., Turner P.E., Chao L. (2005). Widespread genetic exchange among terrestrial bacteriophages. Proc. Natl. Acad. Sci. USA.

[74] Drake J.W. (1993). Rates of spontaneous mutation among RNA viruses. Proc. Natl. Acad. Sci. USA.

[75] Viral Zone: Leviviridae. https://viralzone.expasy.org/163?outline=all_by_species.

[76] Olsthoorn R., Duin J. (2011). van Bacteriophages with ssRNA. eLS.

[77] Zinder N.D. (1965). RNA phages. Annu. Rev. Microbiol..

[78] Dryden S.K., Ramaswami B., Yuan Z., Giammar D.E., Angenent L.T. (2006). A rapid reverse transcription-PCR assay for F+ RNA coliphages to trace fecal pollution in Table Rock Lake on the Arkansas–Missouri border. Water Res..

[79] Cole D., Long S.C., Sobsey M.D. (2003). Evaluation of F+ RNA and DNA Coliphages as Source-Specific Indicators of Fecal Contamination in Surface Waters. Appl. Environ. Microbiol..

[80] Karnik S., Billeter M. (1983). The lysis function of RNA bacteriophage Qβ is mediated by the maturation (A2) protein. EMBO J..

[81] Young R. (1992). Bacteriophage Lysis: Mechanism and Regulation. Microbiol. Rev..

[82] Chamakura K.R., Edwards G.B., Young R. (2017). Mutational analysis of the MS2 lysis protein L. Microbiology.

[83] Roberts J.W., Steitz J.E. (1967). The reconstitution of infective bacteriophage R17. Proc. Natl. Acad. Sci. USA.

[84] Krahn P.M., O’Callaghan R.J., Paranchych W. (1972). Stages in phage R17 infection. VI. Injection of A protein and RNA into the host cell. Virology.

[85] Shiba T., Suzuki Y. (1981). Localization of A protein in the RNA-A protein complex of RNA phage MS2. Biochim. Biophys. Acta BBA-Nucleic Acids Protein Synth..

[86] Zechner E.L., Lang S., Schildbach J.F. (2012). Assembly and mechanisms of bacterial type IV secretion machines. Philos. Trans. R. Soc. B Biol. Sci..

[87] Eigen M., Biebricher C.K., Gebinoga M., Gardiner W.C. (1991). The hypercycle. Coupling of RNA and protein biosynthesis in the infection cycle of an RNA bacteriophage. Biochemistry.

[88] Friedman S.D., Genthner F.J., Gentry J., Sobsey M.D., Vinjé J. (2009). Gene Mapping and Phylogenetic Analysis of the Complete Genome from 30 Single-Stranded RNA Male-Specific Coliphages (Family Leviviridae). J. Virol..

[89] Wahba A.J., Miller M.J., Niveleau A., Landers T.A., Carmichael G.G., Weber K., Hawley D.A., Slobin L.I. (1974). Subunit I of Qβ Replicase and 30 S Ribosomal Protein Sl of *Escherichia coli* EVIDENCE FOR THE IDENTITY OF THE TWO PROTEINS. J. Biol. Chem..

[90] Blumenthal T., Landers T.A., Weber K. (1972). Bacteriophage Qβ Replicase Contains the Protein Biosynthesis Elongation Factors EF Tu and EF Ts. Proc. Natl. Acad. Sci. USA.

[91] Agirrezabala X., Frank J. (2009). Elongation in translation as a dynamic interaction among the ribosome, tRNA, and elongation factors EF-G and EF-Tu. Q. Rev. Biophys..

[92] Schmeing T.M., Voorhees R.M., Kelley A.C., Gao Y.-G., Murphy F.V., Weir J.R., Ramakrishnan V. (2009). The Crystal Structure of the Ribosome Bound to EF-Tu and Aminoacyl-tRNA. Science.

[93] Schuette J.-C., Murphy F.V., Kelley A.C., Weir J.R., Giesebrecht J., Connell S.R., Loerke J., Mielke T., Zhang W., Penczek P.A. (2009). GTPase activation of elongation factor EF-Tu by the ribosome during decoding. EMBO J..

[94] Kamen R., Kondo M., Römer W., Weissmann C. (1972). Reconstitution of Qβ Replicase Lacking Subunit α with Protein-Synthesis-Interference Factor i. Eur. J. Biochem..

[95] Meyer F., Weber H., Weissmann C. (1981). Interactions of Qβ replicase with Qβ RNA. J. Mol. Biol..

[96] Schuppli D., Miranda G., Qiu S., Weber H. (1998). A branched stem-loop structure in the M-site of bacteriophage Qβ RNA is important for template recognition by Qβ replicase holoenzyme. J. Mol. Biol..

[97] Miranda G., Schuppli D., Barrera I., Hausherr C., Sogo J.M., Weber H. (1997). Recognition of bacteriophage Qβ plus strand RNA as a template by Qβ replicase: Role of RNA interactions mediated by ribosomal proteins S1 and host factor1. J. Mol. Biol..

[98] Schuppli D., Georgijevic J., Weber H. (2000). Synergism of mutations in bacteriophage qβ RNA affecting host factor dependence of qβ replicase1. J. Mol. Biol..

[99] Robertson H., Webster R.E., Zinder N.D. (1968). Bacteriophage Coat Protein as Repressor. Nature.

[100] Valegrad K., Murray J.B., Stockley P.G., Stonehouse N.J., Liljas L. (1994). Crystal structure of an RNA bacteriophage coat protein-operator complex. Nature.

[101] Bernhardt T.G., Wang I.-N., Struck D.K., Young R. (2002). Breaking free: “Protein antibiotics” and phage lysis. Res. Microbiol..

[102] Walderich B., Höltje J.V. (1989). Specific localization of the lysis protein of bacteriophage MS2 in membrane adhesion sites of *Escherichia coli*. J. Bacteriol..

[103] Beremand M.N., Blumenthal T. (1979). Overlapping genes in RNA phage: A new protein implicated in lysis. Cell.

[104] Goessens W.H., Driessen A.J., Wilschut J., van Duin J. (1988). A synthetic peptide corresponding to the C-terminal 25 residues of phage MS2 coded lysis protein dissipates the protonmotive force in Escherichia coli membrane vesicles by generating hydrophilic pores. EMBO J..

[105] Walderich B., Ursinus-Wössner A., van Duin J., Höltje J.V. (1988). Induction of the autolytic system of Escherichia coli by specific insertion of bacteriophage MS2 lysis protein into the bacterial cell envelope. J. Bacteriol..

[106] Chamakura K.R., Tran J.S., Young R. (2017). MS2 Lysis of Escherichia coli Depends on Host Chaperone DnaJ. J. Bacteriol..

[107] Lodish H.F. (1970). Secondary structure of bacteriophage f2 ribonucleic acid and the initiation of in vitro protein biosynthesis. J. Mol. Biol..

[108] Kozak M. (1983). Comparison of initiation of protein synthesis in procaryotes, eucaryotes, and organelles. Microbiol. Rev..

[109] Poot R.A., Tsareva N.V., Boni I.V., van Duin J. (1997). RNA folding kinetics regulates translation of phage MS2 maturation gene. Proc. Natl. Acad. Sci. USA.

[110] Weber H. (1976). The binding site for coat protein on bacteriophage Qβ RNA. Biochim. Biophys. Acta BBA-Nucleic Acids Protein Synth..

[111] Groeneveld H., Thimon K., van Duin J. (1995). Translational control of maturation-protein synthesis in phage MS2: A role for the kinetics of RNA folding?. RNA.

[112] Reed C.A., Langlais C., Wang I.-N., Young R. (2013). A2 expression and assembly regulates lysis in Qβ infections. Microbiology.

[113] Rumnieks J., Tars K. (2017). Crystal Structure of the Maturation Protein from Bacteriophage Qβ. J. Mol. Biol..

[114] Atkins J.F., Steitz J.A., Anderson C.W., Model P. (1979). Binding of mammalian ribosomes to ms2 phage RNA reveals an overlapping gene encoding a lysis function. Cell.

[115] Berkhout B., Schmidt B.F., van Strien A., van Boom J., van Westrenen J., van Duin J. (1987). Lysis gene of bacteriophage MS2 is activated by translation termination at the overlapping coat gene. J. Mol. Biol..

[116] Schmidt B.F., Berkhout B., Overbeek G.P., van Strien A., van Duin J. (1987). Determination of the RNA secondary structure that regulates lysis gene expression in bacteriophage MS2. J. Mol. Biol..

[117] Khazaie K., Buchanan J.H., Rosenberger R.F. (1984). The accuracy of Qbeta RNA translation. 1. Errors during the synthesis of Qbeta proteins by intact Escherichia coli cells. Eur. J. Biochem..

[118] Weiner A.M., Weber K. (1971). Natural read-through at the UGA termination signal of Q-beta coat protein cistron. Nat. New Biol..

[119] Robertson H.D., Lodish H.F. (1970). Messenger Characteristics of Nascent Bacteriophage RNA. Proc. Natl. Acad. Sci. USA.

[120] Klovins J., Overbeek G.P., van den Worm S.H.E., Ackermann H.-W., van Duin J. (2002). Nucleotide sequence of a ssRNA phage from Acinetobacter: Kinship to coliphages. J. Gen. Virol..

[121] Bradley D.E. (1966). The Structure and Infective Process of a Pseudomonas Aeruginosa Bacteriophage Containing Ribonucleic Acid. J. Gen. Microbiol..

[122] Olsen R.H., Thomas D.D. (1973). Characteristics and Purification of PRR1, an RNA Phage Specific for the Broad Host Range Pseudomonas R1822 Drug Resistance Plasmid. J. Virol..

[123] Olsthoorn R.C.L., Garde G., Dayhuff T., Atkins J.F., Van Duin J. (1995). Nucleotide sequence of a single-stranded RNA phage from Pseudomonas aeruginosa: Kinship to coliphages and conservation of regulatory RNA structures. Virology.

[124] Ruokoranta T.M., Grahn A.M., Ravantti J.J., Poranen M.M., Bamford D.H. (2006). Complete Genome Sequence of the Broad Host Range Single-Stranded RNA Phage PRR1 Places It in the Levivirus Genus with Characteristics Shared with Alloleviviruses. J. Virol..

[125] Weissmann C., Billeter M.A., Goodman H.M., Hindley J., Weber H. (1973). Structure and Function of Phage RNA. Annu. Rev. Biochem..

[126] Gottesman S., Storz G. (2015). RNA reflections: Converging on Hfq. RNA.

[127] Schuppli D., Miranda G., Tsui H.-C.T., Winkler M.E., Sogo J.M., Weber H. (1997). Altered 3′-terminal RNA structure in phage Qβ adapted to host factor-less Escherichia coli. Proc. Natl. Acad. Sci. USA.

[128] Weber H., Weissmann C. (1970). The 3’-Termini of Bacteriophage Qβ Plus and Minus Strands. J. Mol. Biol..

[129] Blumenthal T., Carmichael G.G. (1979). RNA replication: Function and structure of Qbeta-replicase. Annu. Rev. Biochem..

[130] Bausch J.N., Kramer F.R., Miele E.A., Dobkin C., Mills D.R. (1983). Terminal Adenylation in the Synthesis of RNA by Qβ Replicase. J. Biol. Chem..

[131] Beekwilder J., Nieuwenhuizen R., Poot R. (1996). Secondary Structure Model for the First Three Domains of Qβ RNA. Control of A-protein synthesis. J. Mol. Biol..

[132] Staples D.H., Hindley J., Billeter M.A., Weissmann C. (1971). Localization of Qβ Maturation Cistron Ribosome Binding Site. Nat. New Biol..

[133] Rumnieks J., Tars K. (2011). Crystal structure of the read-through domain from bacteriophage Qβ A1 protein. Protein Sci..

[134] Kastelein R.A., Remaut E., Fiers W., Duin J. (1982). van Lysis gene expression of RNA phage MS2 depends on a frameshift during translation of the overlapping coat protein gene. Nature.

[135] Bernhardt T.G., Wang I.-N., Struck D.K., Young R. (2001). A Protein Antibiotic in the Phage Qβ Virion: Diversity in Lysis Targets. Science.

[136] Brown E.D., Vivas E.I., Walsh C.T., Kolter R. (1995). MurA (MurZ), the enzyme that catalyzes the first committed step in peptidoglycan biosynthesis, is essential in Escherichia coli. J. Bacteriol..

[137] Grasis J.A., Pantaleo V., Chiumenti M. (2018). Host-Associated Bacteriophage Isolation and Preparation for Viral Metagenomics. Viral Metagenomics.

[138] Krishnamurthy S.R., Wang D. (2018). Extensive conservation of prokaryotic ribosomal binding sites in known and novel picobirnaviruses. Virology.

[139] Adriaenssens E., Farkas K., Harrison C., Jones D., Allison H.E., McCarthy A.J. (2018). Viromic analysis of wastewater input to a river catchment reveals a diverse assemblage of RNA viruses. MSystems.

[140] Lister P.D., Wolter D.J., Hanson N.D. (2009). Antibacterial-Resistant Pseudomonas aeruginosa: Clinical Impact and Complex Regulation of Chromosomally Encoded Resistance Mechanisms. Clin. Microbiol. Rev..

[141] Kim E.S., Bae H.-W., Cho Y.-H. (2018). A Pilin Region Affecting Host Range of the Pseudomonas aeruginosa RNA Phage, PP7. Front. Microbiol..

